# Associations between sex, body mass index and the individual microglial response in Alzheimer’s disease

**DOI:** 10.1186/s12974-024-03020-y

**Published:** 2024-01-23

**Authors:** Gloria Biechele, Boris-Stephan Rauchmann, Daniel Janowitz, Katharina Buerger, Nicolai Franzmeier, Endy Weidinger, Selim Guersel, Sebastian Schuster, Anika Finze, Stefanie Harris, Simon Lindner, Nathalie L. Albert, Christian Wetzel, Rainer Rupprecht, Axel Rominger, Carla Palleis, Sabrina Katzdobler, Lena Burow, Carolin Kurz, Mirlind Zaganjori, Lena-Katharina Trappmann, Oliver Goldhardt, Timo Grimmer, Jan Haeckert, Daniel Keeser, Sophia Stoecklein, Estrella Morenas-Rodriguez, Peter Bartenstein, Johannes Levin, Günter U. Höglinger, Mikael Simons, Robert Perneczky, Matthias Brendel

**Affiliations:** 1 Department of Nuclear Medicine, LMU University Hospital, LMU Munich, University of Munich, Marchioninstraße 15, 81377 Munich, Germany; 2 Department of Radiology, LMU University Hospital, LMU Munich, Munich, Germany; 3 Institute for Stroke and Dementia Research, LMU University Hospital, LMU Munich, Munich, Germany; 4 German Center for Neurodegenerative Diseases (DZNE), Munich, Germany; 5 Department of Neurology, LMU University Hospital, LMU Munich, Munich, Germany; 6 Department of Psychiatry and Psychotherapy, LMU University Hospital, LMU Munich, Munich, Germany; 7 Institute of Neuroradiology, LMU University Hospital, LMU Munich, Munich, Germany; 8 Department of Psychiatry and Psychotherapy, University of Regensburg, Regensburg, Germany; 9 Department of Nuclear Medicine, University of Bern, Inselspital, Bern, Switzerland; 10 Department of Psychiatry and Psychotherapy, School of Medicine and Health, Technical University Munich, Klinikum Rechts Der Isar, Munich, Germany; 11 Department of Psychiatry, Psychotherapy and Psychosomatics, Medical Faculty, University of Augsburg, Augsburg, Germany; 12 Munich Cluster for Systems Neurology (SyNergy), Munich, Germany; 13 Institute of Neuronal Cell Biology, TU Munich, Munich, Germany; 14 Sheffield Institute for Translational Neuroscience (SITraN), University of Sheffield, Sheffield, UK; 15 Department of Psychiatry and Neurochemistry, The Sahlgrenska Academy, Institute of Neuroscience and Physiology, University of Gothenburg, Mölndal, Gothenburg, Sweden; 16 Ageing Epidemiology (AGE) Research Unit, School of Public Health, Imperial College London, London, UK

**Keywords:** Microglia, TSPO, Amyloid, Tau, Sex differences

## Abstract

**Background and objectives:**

18-kDa translocator protein position-emission-tomography (TSPO-PET) imaging emerged for in vivo assessment of neuroinflammation in Alzheimer’s disease (AD) research. Sex and obesity effects on TSPO-PET binding have been reported for cognitively normal humans (CN), but such effects have not yet been systematically evaluated in patients with AD. Thus, we aimed to investigate the impact of sex and obesity on the relationship between β-amyloid-accumulation and microglial activation in AD.

**Methods:**

49 patients with AD (29 females, all Aβ-positive) and 15 Aβ-negative CN (8 female) underwent TSPO-PET ([^18^F]GE-180) and β-amyloid-PET ([^18^F]flutemetamol) imaging. In 24 patients with AD (14 females), tau-PET ([^18^F]PI-2620) was additionally available. The brain was parcellated into 218 cortical regions and standardized-uptake-value-ratios (SUVr, cerebellar reference) were calculated. Per region and tracer, the regional increase of PET SUVr (z-score) was calculated for AD against CN. The regression derived linear effect of regional Aβ-PET on TSPO-PET was used to determine the Aβ-plaque-dependent microglial response (slope) and the Aβ-plaque-independent microglial response (intercept) at the individual patient level. All read-outs were compared between sexes and tested for a moderation effect of sex on associations with body mass index (BMI).

**Results:**

In AD, females showed higher mean cortical TSPO-PET z-scores (0.91 ± 0.49; males 0.30 ± 0.75; *p* = 0.002), while Aβ-PET z-scores were similar. The Aβ-plaque-independent microglial response was stronger in females with AD (+ 0.37 ± 0.38; males with AD − 0.33 ± 0.87; *p* = 0.006), pronounced at the prodromal stage. On the contrary, the Aβ-plaque-dependent microglial response was not different between sexes. The Aβ-plaque-independent microglial response was significantly associated with tau-PET in females (Braak-II regions: *r* = 0.757, *p* = 0.003), but not in males. BMI and the Aβ-plaque-independent microglial response were significantly associated in females (*r* = 0.44, *p* = 0.018) but not in males (BMI*sex interaction: *F*_(3,52)_ = 3.077, *p* = 0.005).

**Conclusion:**

While microglia response to fibrillar Aβ is similar between sexes, women with AD show a stronger Aβ-plaque-independent microglia response. This sex difference in Aβ-independent microglial activation may be associated with tau accumulation. BMI is positively associated with the Aβ-plaque-independent microglia response in females with AD but not in males, indicating that sex and obesity need to be considered when studying neuroinflammation in AD.

**Supplementary Information:**

The online version contains supplementary material available at 10.1186/s12974-024-03020-y.

## Introduction

Alzheimer disease (AD) is the most prevalent neurodegenerative disease in societies with aging populations [1]. The neuropathology of AD is characterized by the histological triad of accumulation of extracellular amyloid-β peptide (Aβ) plaques, intracellular fibrillary tau aggregates within neurons, and the activation of neuroinflammatory pathways mediated by microglia and astrocytes [2–4]. Importantly, microglial activation can be assessed by 18 kDa translocator protein (TSPO) positron-emission-tomography (TSPO-PET) which has received growing interest in the last decade [5]. In this regard, molecular imaging for stratification and monitoring of glial activation could become crucial for target engagement and response assessment of immunomodulatory therapies [6].

Clinical data indicate that men and women exhibit sex differences in the neuropathological and symptomatic progression of AD [7]. The lifetime risk for developing AD for 65-year-old females is twice that of men of the same age (12% vs. 6.3%, respectively) [8]. Furthermore, women show faster progression from mild cognitive impairment to AD dementia when compared to men [9]. There is growing evidence that sex differences in neuroinflammation pathways, including microglial activation, could play a crucial role in driving the sex differences observed in AD [10]. Notably, glial cells express receptors for estrogens and androgens, suggesting the potential for modulation of neuroinflammatory responses by sex steroid hormones [11]. Although detailed functions of TSPO remain to be elucidated, a key pathway attributed to TSPO in microglia is cholesterol transport within the mitochondria [12], which implies downstream effects on the synthesis and metabolism of sex steroid hormones. Indeed, a recent human study in cognitively healthy individuals revealed significant sex differences of [^11^C]PBR28 binding in brain, with women showing a higher TSPO-PET signal [11]. Similarly, we were able to detect a stronger age related increase of the TSPO-PET signal in female wild-type mice studied from 2.5 to 12.5 months of age when compared to male wild-type mice [13]. Furthermore, in an Aβ mouse model, we pinpointed higher levels of the [^18^F]GE-180 TSPO-PET signal and microglial immunohistochemistry markers in female *App*^*NL−G−F*^ mice when compared to their male littermates [13, 14]. Even more important, sex determined the response to an immunomodulatory PPARγ agonist therapy, showing TSPO-PET reductions only in female *App*^*NL−G−F*^ mice [14]. When in addition considering tau, there is evidence for a reciprocal relationship between microglial activation, β-amyloid burden and tau accumulation in females, whereas the effects of microglial activation and β-amyloid on tau are more independent in males [15]. To shed light of these histopathological findings in vivo, we added tau-PET as a sub-item to our analyses, when available, since we found tau to be the strongest ATN predictor to neuroinflammation in primary and secondary tauopathies [16].

Overall, we aimed to investigate sex differences in TSPO-PET imaging of a human AD cohort. To this end, we analyzed regional Aβ-PET and TSPO-PET signals to determine the Aβ-plaque dependent and the Aβ-plaque-independent microglial response in comparison of females and males with AD. Furthermore, we investigated the associations between Aβ-plaque dependent and independent microglial responses with tau accumulation as well as the interaction of sex, body mass index (BMI) and microglial activation.

## Material and methods

### Study design and patient cohort

The data used in this study originate from the baseline dataset of the ActiGliA study, a prospective, longitudinal, observational, single-center study of the Munich Cluster for Systems Neurology (SyNergy) at Ludwig-Maximilians-University (LMU) Munich, initiated in 2017 [17]. Participants were recruited through specialized outpatient clinics at the LMU hospital Department of Psychiatry and Psychotherapy, Department of Neurology and Institute of Stroke and Dementia Research and the Department of Psychiatry and Psychotherapy of the Technical University Munich. ActiGliA comprises comprehensive neurocognitive, clinical and lifestyle assessments based on the German Center for Neurodegenerative Disorders (DZNE)-Longitudinal Cognitive Impairment and Dementia (DELCODE) study [18]; MRI and PET imaging using tracers for Aβ, TSPO and tau; and fluid biobanking, including CSF, plasma, serum, saliva, DNA, RNA and peripheral blood mononuclear cells. Patients with early AD (subjective cognitive impairment due to AD, MCI due to AD and mild AD dementia) and AD with corticobasal syndrome (AD-CBS) and age-matched healthy controls were included after providing written informed consent in line with the declaration of Helsinki. Clinical diagnosis of CBS was made as defined in the MDS-PSP criteria [19] and AD-CBS was defined by a positive β-amyloid-PET scan. Inclusion and exclusion criteria for the ActiGliA cohort have been reported previously [17, 20]. The study was approved by the ethics committee of LMU Munich (project numbers 17-755 and 17-569).

Out of 140 ActiGliA participants, all cases available 5/2021, meeting the selection criteria for the present analyses were included, resulting in a cohort of 49 patients across the AD continuum (including AD-CBS) and 15 Aβ-negative HC. Selection criteria were (i) available TSPO-PET ([^18^F]GE-180) and (ii) available β-amyloid-PET imaging (Aβ-PET; [^18^F]flutemetamol). AD continuum was defined as CDR global score ≥ 0.5, CERAD-NB total score ≤ 84 and presence of Aβ pathology on PET and/or CSF examination. Cognitively normal controls (CN) were defined as participants without cognitive impairment (Clinical Dementia Rating (CDR) global score = 0, Consortium to Establish a Registry for AD neuropsychological battery (CERAD-NB) total score ≥ 69) and no indication of Aβ pathology on PET (negative visual read) and/or CSF examination (normal Aβ_42/40_-ratio as defined below).

### Clinical assessments

All tests are described elsewhere [17]. In brief, the CDR, CERAD-NB and Mini-Mental State Examination (MMSE) were conducted by trained psychologists at the LMU hospital memory clinic. Using the CERAD-NB battery a total score was created as shown previously, comprising the six sub-tests semantic fluency (animals/60 s), modified Boston Naming Test, Word List Learning, Constructional Praxis, Word List Recall and Word List Recognition Discriminability, with higher scores indicating better performance.

### PET imaging and analysis

*PET data acquisition, reconstruction and post-processing* For all PET procedures, including radiochemistry, acquisition and pre-processing, we used an established and standardized protocol [21]. In brief, [^18^F]GE-180 TSPO-PET recordings (average dose: 177 ± 17 MBq) with an emission window of 60–80 min after injection were obtained to measure glial activation [21]. [^18^F]flutemetamol Aβ-PET recordings (average dose: 182 ± 11 MBq) with an emission window of 90–110 min after injection were performed for assessment of fibrillar Aβ accumulation. Dynamic [^18^F]PI-2620 tau-PET (average dose: 186 ± 14 MBq) with emission recording 0–60 min after injection was performed to quantify tau aggregation. Static frames of the late phase (20–40 min) [22] were reconstructed and used for further processing and analysis.

*PET image analysis* We performed all PET data analyses using PMOD (version 3.9; PMOD technologies). Static emission recordings were coregistered to the Montreal Neurology Institute (MNI) space using non-linear warping (16 iterations, frequency cutoff 25, transient input smoothing 8 × 8 × 8 mm) to tracer specific templates acquired in previous in house studies [21]. Given the strong binding differences of positive and negative Aβ-PET images, we used positive and negative Aβ-PET templates after classification of the Aβ-status by a visual read by a single rater. A unified template was used for TSPO-PET. Intensity normalization of TSPO-, Aβ- and tau-PET images was performed by standardized uptake value ratios (SUVr) using the cerebellum as an established pseudo-reference tissue for TSPO-PET [23]. The cerebellar grey matter was selected as the best compromise of a unified pseudo-reference tissue since it was also validated for [^18^F]flutemetamol [24] and [^18^F]PI-2620. According to Brainnetome atlas [25], the brain was parcellated into 218 cortical regions and standardized-uptake-value-ratios (SUVr) were calculated for TSPO-, Aβ- and tau-PET. Per AD patient, the averaged regional increase of TSPO-, Aβ- and tau-PET SUVr (z-score) was calculated versus CN. For tau-PET, late static quantification was validated against modeling of the 60 min dynamic PET scan (see Additional file 1). A region-based correlation of the 218 cortical TSPO z-scores vs the 218 Aβ z-scores was performed to define a two-dimensional microglia response index per patient. In particular, we used the function between regional Aβ-PET and TSPO-PET SUVr to determine the Aβ-plaque-dependent microglial response (slope) and the Aβ-plaque-independent microglial response (intercept) at the single patient level. Here, we assumed that Aβ-PET predominately detects fibrillar Aβ [26] which implies that the multi-regional TSPO-Aβ correlation of single patient data represents the magnitude of microglia activation to plaques. The resulting Aβ-plaque-independent microglial response (intercept) corresponds to the y-axis intercept of this function and determines the level of TSPO-PET z-score corresponding to zero Aβ-PET z-score alteration, thus implying the magnitude of microglial activation distant/independent from plaques. Furthermore, we calculated composite TSPO-, Aβ- and tau-PET z-scores in commonly evaluated regions of amyloidosis [27] and Braak-stage regions [28]. These amyloidosis (frontal, temporal, parietal, posterior cingulate cortex/ precuneus) and Braak-stage (I–VI) regions were defined by single regions of the Brainnetome atlas.

### Genetic polymorphisms

TSPO genotyping was performed at the Departments of Psychiatry and Psychotherapy of University of Regensburg and LMU Munich, respectively. Genomic DNA was extracted from whole blood using a SQ Blood DNA kit from Omega Bio-Tek (Norcross, GA, USA) according to the manufacturer’s protocol. DNA quality was assessed by optical absorbance and gel electrophoresis. TaqMan quantitative polymerase chain reaction assays were used for amplification and Sanger method for sequencing. Binding affinity of the [^18^F]GE-180 TSPO ligand is affected by the co-dominant rs6971 (Ala/Thr) single nucleotide polymorphisms (SNP) of the TSPO gene and needs to be considered in the imaging analysis [29]. High-affinity binders (HAB) are Ala/Ala carriers, low-affinity binders (LAB) are Thr/Thr carriers and mixed-affinity binders (MAB) are Ala/Thr carriers. Only HAB and MAB carriers were included in the PET analyses, with *N* = 6 LAB excluded. All TSPO-PET analyses were adjusted for binding status.

### CSF analyses

CSF peptide measures were generated from aliquoted samples using commercially available (Fujirebio, Malvern, PA) enzyme-linked immunosorbent assays (ELISAs). Aβ positivity was defined as a CSF Aβ_42/40_-ratio of < 5.5%, as suggested previously [30]. Concentrations of total tau (ttau) and *p*-tau-181 (normal range: < 61 pg/ml) were measured in CSF using the Innotest htau-Ag, and Innotest P-tau (181P) ELISA assays (Fujirebio, Europe) and the ttau/Aβ index (1.18 × ttau Aβ + 1240; normal range < 1000) was used as an AD specific index of neuronal injury.

### Statistics

For statistical calculations, SPSS (V25; IBM; Armonk, New York, USA) and GraphPad Prism (V9, Boston, Massachusetts USA) were used.

To test for group differences in demographic data, an unpaired two-tailed Student’s *t*-test was applied. An equal distribution of clinical presentation in the AD group was confirmed by a Chi-square (*χ*^2^) test.

We tested for a sex effect between regional TSPO- and Aβ-PET *z*-scores using an univariate analysis of variance (ANCOVA), controlling for age, BMI and the TSPO gene SNP as well as applying false discovery rate (FDR) correction for multiple brain regions (four regions of amyloidosis and six Braak-stage regions).

For each individual subject, a linear regression between regional Aβ-PET SUVr and TSPO-PET SUVr was used to determine the Aβ-plaque-dependent microglial response (slope, β) and the Aβ-plaque-independent microglial response (intercept). Afterwards, Aβ-independent (intercept) and -dependent (β) microglial activation was compared between females and males with AD, cognitively normal female and males and patients and controls per sex-related group by univariate analysis of variance (ANCOVA), controlling for age, BMI and the TSPO gene SNP. Due to stage-dependent elevations of TSPO-PET binding, a dedicated comparison of females and males in prodromal (SCD and MCI) and AD dementia (ADD, AD-CBS) patients was performed analogously. FDR correction for multiple comparisons was applied.

In a partial correlation analysis, we tested if tau-PET z-scores in Braak-stage regions explain Aβ-independent microglial activation, controlled for age, BMI and the TSPO gene SNP. Additionally a partial correlation, controlled for age, BMI and the TSPO gene SNP, was also used to test if fluid biomarkers (Aβ_42/40_ ratio, p-tau-181, tau/Aβ index) explain elevated Aβ-plaque-independent TSPO-PET signals in females with AD.

When finally examining an association between age or BMI and the Aβ-independent microglial activation in the AD patient cohort, a linear regression analysis corrected for the TSPO gene SNP was applied. Furthermore we used a linear regression to test for an age*sex interaction and a BMI*sex interaction controlled for age or BMI, TSPO gene SNP and AD signature CSF markers (Aβ_42/40_ ratio, p-tau-181, tau/Aβ index). This analysis was repeated for the Aβ-plaque associated microglial response as the outcome variable. Statistical parametric mapping (SPM, V12, Wellcome Centre for Human Neuroimaging, London, UK) [17] was complimented to perform the linear regression analysis on the voxel-level. The SPM analysis was corrected for multiple comparisons by a false discovery rate (FDR) correction [31]. To avoid bias by regional constitution of single voxels, we used the Brainnetome atlas regions to obtain a topological t-score threshold for FDR correction [32]. An FDR-corrected *p*-value of 0.05 was set as the threshold for voxel-wise analyses.

For all models, we applied an alpha threshold of 0.05 for considering effects to be statistically significant.

## Results

### Demographics

The analyzed cohort included 49 patients with AD and 15 controls (Table 1). Age (69.6 ± 8.4 vs. 73.4 ± 5.6, *p* = 0.12), BMI (24.5 ± 4.5 vs. 25.2 ± 3.0, *p* = 0.41) and the TSPO gene SNP (HAB 12/29; MAB 17/29 vs. HAB 11/20; MAB 9/20; *χ*^2^ = 0.88, *p* = 0.35) did not differ between females and males with AD. The distribution of clinical presentation of prodromal (SCD, MCI) and manifest (ADD, AD-CBS) AD did not differ between males and females (*χ*^2^ = 1.89, *p* = 0.60).

**Table 1:** Demographics, clinical cognitive assessment scores, clinical presentation, Aβ-positivity, CSF levels and single nucleotide polymorphism of the TSPO gene at the group level

Demographics	Alzheimer’s disease			Controls		
	All	Female	Male	All	Female	Male
*n*	49	29	20	15	8	7
Age	71.1 ± 7.5 (68.9–73.3)	69.6 ± 8.2 (66.4–72.7)	73.4 ± 5.8 (70.6–76.2)	70.7 ± 7.5 (66.9–74.6)	68.9 ± 8.0 (61.8–76.0)	72.8 ± 4.0 (68.8–76.7)
BMI	24.8 ± 4.1 (23.6–26.0)	24.5 ± 4.7 (22.7–26.3)	25.2 ± 2.9 (23.8–26.6)	25.0 ± 3.5 (23.0–27.0)	23.6 ± 3.4 (20.6–26.6)	26.6 ± 3.0 (23.6–29.6)
MMSE	24.2 ± 5.2 (22.7–25.6)	24.4 ± 4.2 (22.7–25.9)	23.9 ± 6.2 (20.9–26.9)	29.1 ± 1.0 (28.6–29.7)	29.0 ± 1.0 (28.1–29.9)	29.3 ± 0.9 (28.4–30.2)
CERAD	4.5 ± 2.9 (3.6–5.5)	5.0 ± 2.9 (3.7–6.4)	3.9 ± 2.9 (2.3–5.5)	0.4 ± 0.6 (0.1–0.8)	0.6 ± 0.7 (0.0–1.3)	0.1 ± 0.3 (− 0.2–0.5)
CDR (sob)	3.2 ± 2.3 (2.6–3.9)	3.4 ± 2.3 (2.6–4.3)	2.9 ± 2.3 (1.8–4.0)	0.1 ± 0.2 (0.0–0.2)	0.1 ± 0.2 (− 0.1–0.2)	0.1 ± 0.2 (− 0.1–0.4)
SCD/ MCI/ ADD/ atyp	7 / 12 / 18 / 12	3 / 6 / 12 / 8	4 / 6 / 6 / 4	15 / 0 / 0 / 0	8 / 0 / 0 / 0	7 / 0 / 0 / 0
Aβ-PET positivity (%)	100%	100%	100%	0%	0%	0%
CSF Aβ_42_ (pg/ml)	528 ± 188 (465–592)	595 ± 176 (515–674)	431 ± 161 (338–523)	1008 ± 641 (605–1411)	1243 ± 642 (505–1981)	807 ± 567 (241–1373)
CSF Aβ_40_ (pg/ml)	13,506 ± 5767 (11,557–15,456)	14,985 ± 4904 (12,759–17,210)	11,338 ± 6234 (7764–14,912)	13,748 ± 6925 (9392–18,103)	16,272 ± 5240 (10,248–22,295)	11,584 ± 7442 (4149–19,018)
CSF Aβ_42/40_ ratio	4.2 ± 1.2 (3.8–4.6)	4.1 ± 1.1 (3.6–4.6)	4.2 ± 1.4 (3.4–5.0)	7.0 ± 1.6 (6.0–8.0)	7.3 ± 1.3 (5.7–8.8)	6.8 ± 1.8 (5.0–8.5)
CSF t-Tau (pg/ml)	528 ± 536 (350–707)	535 ± 197 (447–622)	519 ± 817 (51–987)	299 ± 143 (204–394)	313 ± 179 (108–518)	285 ± 92 (179–391)
CSF p-Tau (pg/ml)	85 ± 42 (71–99)	89 ± 24 (78–100)	79 ± 60 (43–114)	56 ± 16 (45–67)	56 ± 18 (36–76)	56 ± 15 (39–72)
CSF Tau/Aβ index	1336 ± 632 (1120–1553)	1276 ± 283 (1144–1407)	1422 ± 913 (898–1945)	595 ± 303 (311–879)	497 ± 139 (80–914)	693 ± 382 (160–1227)
rs6971 SNP	HAB: 22 / MAB: 27	HAB: 11 / MAB: 18	HAB: 11 / MAB: 9	HAB: 3 / MAB: 12	HAB: 1 / MAB: 7	HAB: 2 / MAB: 5
Time between Aβ- and TSPO-PET (m)	2.0 ± 3.9 (0.8–3.1)	2.3 ± 4.8 (0.5–4.2)	1.5 ± 1.8 (0.6–2.3)	0.1 ± 0.3 (− 0.1–0.2)	0.2 ± 0.2 (0.1–0.3)	0.1 ± 0.4 (− 0.2–0.5)

Mean ± standard deviation (95%-CI)

### Females with AD show higher TSPO-PET signals at similar levels of fibrillar Aβ

**Fig. 1:**
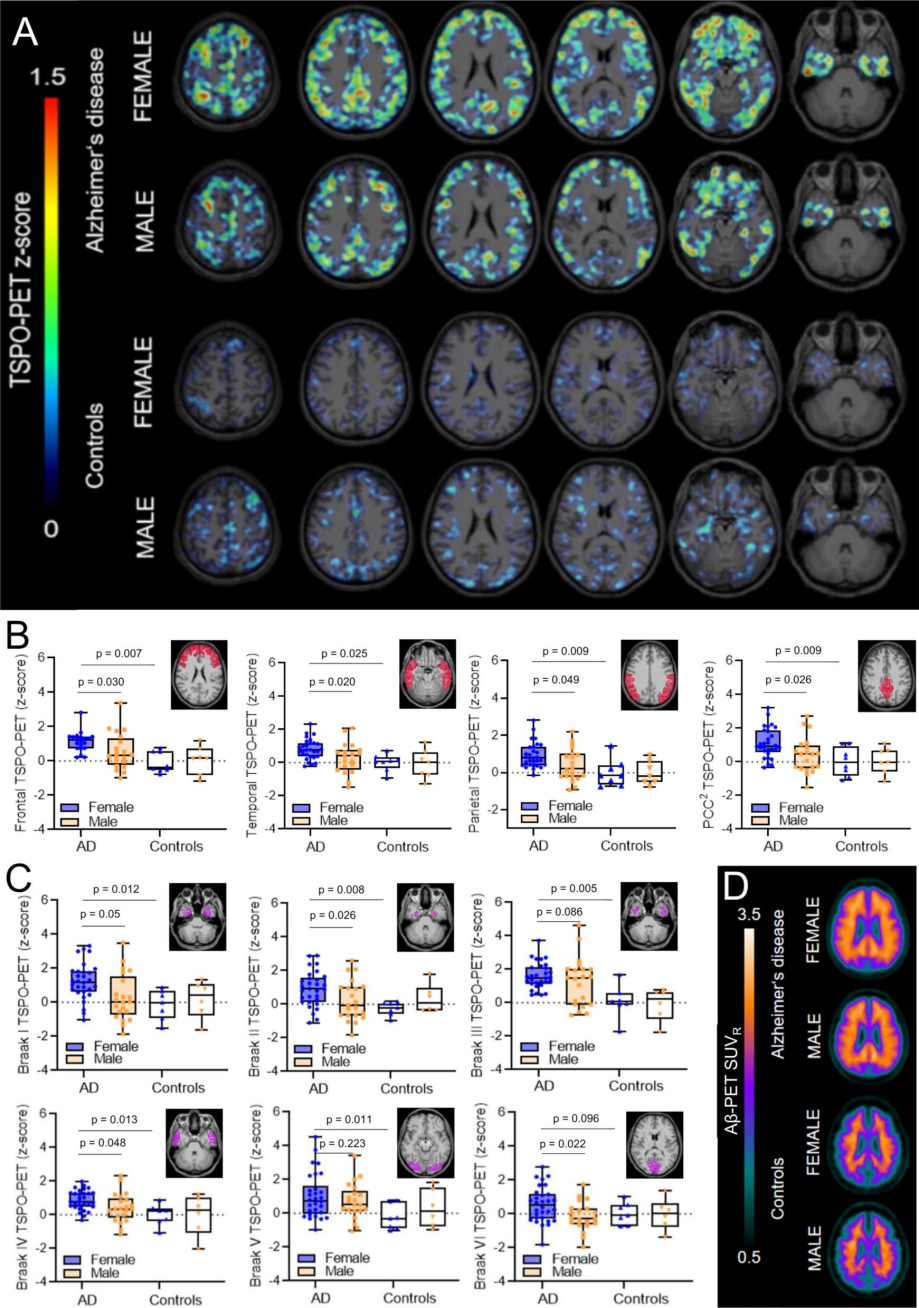
[^18^F]GE-180 TSPO-PET binding in predefined target regions. **A** Group levels of [^18^F]GE-180 TSPO-PET z-scores (cerebellum scaled standardized uptake value ratio, SUV_R_) between sexes in AD patients and cognitively normal controls, presented as axial overlays upon a standard magnetic resonance imaging template. **B** TSPO-PET *z*-scores in the comparison of AD diagnosis group vs. cognitively normal males and females for frontal, temporal, parietal and posterior cingulate cortex/ precuneus (PCC^2^). Anatomic regions are shown upon an axial MRI atlas. **C** TSPO-PET z-scores in the comparison of AD diagnosis group vs. cognitively normal males and females for Braak-stage I–VI regions. Anatomic regions are shown upon an axial MRI atlas. **D** Axial images show group levels of [^18^F]flutemetamol Aβ-PET (cerebellum scaled standardized uptake value ratio, SUV_R_) in AD and cognitively normal controls in comparison between sexes, projected upon a standard MRI anatomic template. AD female *n* = 29, AD male *n* = 20, CN female *n* = 8, CN male *n* = 7

First, we applied typical target regions of amyloidosis [27] and Braak-stages [28] as predefined regions of interest to TSPO- and Aβ-PET analyses. Here, females with AD indicated higher TSPO-PET *z*-scores when compared to males with AD in the frontal cortex (1.21 ± 0.15 vs. 0.51 ± 0.19, *p* = 0.030), the temporal cortex (0.81 ± 0.14 vs. 0.18 ± 0.18, *p* = 0.020), the parietal cortex (0.92 ± 0.14 vs. 0.43 ± 0.18, *p* = 0.049), posterior cingulate cortex/precuneus (1.13 ± 0.18 vs. 0.39 ± 0.22, *p* = 0.026) and in the Braak-stage II (0.97 ± 0.21 vs. 0.21 ± 0.26, *p* = 0.026), Braak-stage IV (0.92 ± 0.13 vs. 0.43 ± 0.16, *p* = 0.048) as well as Braak-stage VI (0.64 ± 0.18 vs. − 0.17 ± 0.22, *p* = 0.022) regions of interest, after controlling for age, BMI and TSPO gene SNP as well as for multiple comparisons (Fig. 1, Table 2). Females with AD had significantly higher frontal, parietal and posterior cingulate cortex/ precuneus as well as Braak-stage I, Braak-stage II, Braak-stage III, Braak-stage IV and Braak-stage V TSPO-PET z-scores when compared to female controls, whereas males with AD only comprised higher Braak-stage III TSPO-PET z-scores in the contrast against male controls (Fig. 1, Table 2). Composite Aβ-PET z-scores were similar between females and males with AD (6.31 ± 0.37 vs. 6.48 ± 0.45, *p* = 0.77) and cognitively healthy females and males (− 0.17 ± 0.72 vs. 0.39 ± 0.75, *p* = 0.60) after controlling for age, BMI and TSPO gene SNP. Direct consideration of composite Aβ-PET *z*-scores as a covariate in a TSPO-PET subanalysis of AD patients revealed a consistent sex effect between females and males (i.e., TSPO-PET *z*-scores in the frontal cortex: 1.28 ± 0.17 vs. 0.58 ± 0.21, *p* = 0.010). Direct comparisons of medium and high affinity binding status, as determined by the TSPO SNP, did not show any significant differences of TSPO-PET *z*-scores in females and males with AD (Additional file 1: Table S1), fitting to our previous observation of similar [^18^F]GE-180 TSPO-PET signals between subjects with medium and high affinity binding status [33]. Aβ-PET z-scores of females and males with AD were also similar between medium and high affinity binding status (Additional file 1: Table S1). In summary, we observed a robust sex effect, revealing that females comprise higher TSPO-PET levels in typical target regions of AD.

**Table 2:** Detailed regional *z*-scores of TSPO-PET and Aβ-PET at the group level in six Braak-stage regions of interest and four amyloidosis regions of interest

PET	AD			Controls		
TSPO-PET *z*-scores	Female (mean, 95%-CI)	Male (mean, 95%-CI)	*P* ♂/♀ (FDR)	Female (mean, 95%-CI)	Male (mean, 95%-CI)	*P* ♂/♀ (FDR)
Braak I	1.290 (0.846–1.733)^#^	0.252 (− 0.293–0.797)	0.051	− 0.018 (− 0.883–0.847)	0.143 (− 0.767–1.052)	> 0.999
Braak II	0.972 (0.589–1.354)^#^^#^	0.205 (− 0.265–0.675)	0.026*	− 0.265 (− 1.011–0.480)	0.273 (− 0.511–1.057)	> 0.999
Braak III	1.726 (1.344–2.108)^##^	1.175 (0.706–1.644)^#^	0.086	0.294 (− 0.451–1.038)	− 0.231 (− 1.014–0.552)	> 0.999
Braak IV	0.924 (0.642–1.206)	0.431 (0.085–0.778)	0.048*	0.115 (− 0.435–0.665)	− 0.149 (− 0.727–0.429)	> 0.999
Braak V	1.125 (0.689–1.561)^#^	0.693 (0.157–1.229)	0.223	− 0.320 (− 1.170–0.531)	0.132 (− 0.762–1.026)	> 0.999
Braak VI	0.643 (0.284–1.002)	− 0.171 (− 0.612–0.271)	0.022*	− 0.015 (− 0.716–0.685)	0.140 (− 0.596–0.876)	> 0.999
Frontal cortex	1.210 (0.903–1.517)^##^	0.506 (0.129–0.883)	0.030*	0.015 (− 0.584–0.613)	0.059 (− 0.570–0.689)	> 0.999
Temporal cortex	0.814 (0.527–1.101)^#^	0.179 (− 0.173–0.532)	0.020*	0.081 (− 0.479–0.641)	− 0.091 (− 0.680–0.497)	> 0.999
Parietal cortex	0.918 (0.632–1.204)^##^	0.433 (0.082–0.785)	0.049*	− 0.029 (− 0.587–0.530)	0.001 (− 0.586–0.587)	0.943
PCC/precuneus	1.126 (0.768–1.485)^##^	0.391 (− 0.049–0.831)	0.026*	0.010 (− 0.689–0.708)	0.083 (− 0.651–0.818)	> 0.999

Aβ-PET *z*-scores	Female (mean, 95%-CI)	Male (mean, 95%-CI)	*P* ♂/♀ (FDR)	Female (mean, 95%-CI)	Male (mean, 95%-CI)	*P* ♂/♀ (FDR)
Braak I	2.122 (1.528–2.717)^#^^#^	1.976 (1.246–2.706)^#^	0.950	− 0.131 (− 1.290–1.028)	0.247 (− 0.972–1.466)	> 0.999
Braak II	0.801 (0.366–1.235)	0.542 (0.008–1.076)	> 0.999	− 0.133 (− 0.980–0.714)	0.127 (− 0.764–1.018)	> 0.999
Braak III	2.884 (2.288–3.481)^#^^#^^#^	3.109 (2.376–3.841)^#^^#^^#^	0.916	− 0.110 (− 1.272–1.053)	0.155 (− 1.068–1.378)	0.946
Braak IV	5.581 (4.813–6.348)^#^^#^^#^	5.984 (5.005–6.891)^#^^#^^#^	> 0.999	− 0.098 (− 1.594–1.399)	0.334 (− 1.239–1.907)	0.992
Braak V	2.699 (2.111–3.288)^#^^#^^#^	3.233 (2.510–3.956)^#^^#^^#^	> 0.999	0.102 (− 1.045–1.250)	0.023 (− 1.183–1.230)	0.925
Braak VI	3.911 (3.015–4.806)^#^^#^^#^	4.623 (3.523–5.723)^#^^#^	> 0.999	− 0.598 (− 2.344–1.148)	0.787 (− 1.049–2.623)	> 0.999
Frontal cortex	6.121 (5.381–6.861)^#^^#^^#^	5.972 (5.062–6.881)^#^^#^^#^	0.892	− 0.164 (− 1.607–1.279)	0.336 (− 1.181–1.854)	> 0.999
Temporal cortex	5.139 (4.402–5.876)^#^^#^^#^	5.458 (4.552–6.364)^#^^#^^#^	0.987	− 0.216 (− 1.654–1.221)	0.329 (− 1.182–1.841)	> 0.999
Parietal cortex	5.669 (4.962–6.375)^#^^#^^#^	5.717 (4.848–6.585)^#^^#^^#^	0.933	− 0.039 (− 1.417–1.339)	0.178 (− 1.271–1.627)	0.923
PCC/precuneus	8.302 (7.341–9.263)^#^^#^^#^	8.789 (7.608–9.971)^#^^#^^#^	> 0.999	− 0.239 (− 2.114–1.637)	0.723 (− 1.248–2.694)	> 0.999

CI = 95% confidence interval. *P* values show false discovery rate (FDR) corrected significance levels for the comparison of females and males (ANOVA). * *p* < 0.05 for comparison of females and males with AD. ^#^ *p* < 0.05, ^##^ *p* < 0.01, ^###^ *p* < 0.005 for comparison of patients with AD vs. controls

### Females comprise a stronger Aβ-plaque-independent microglial response

**Fig. 2:**
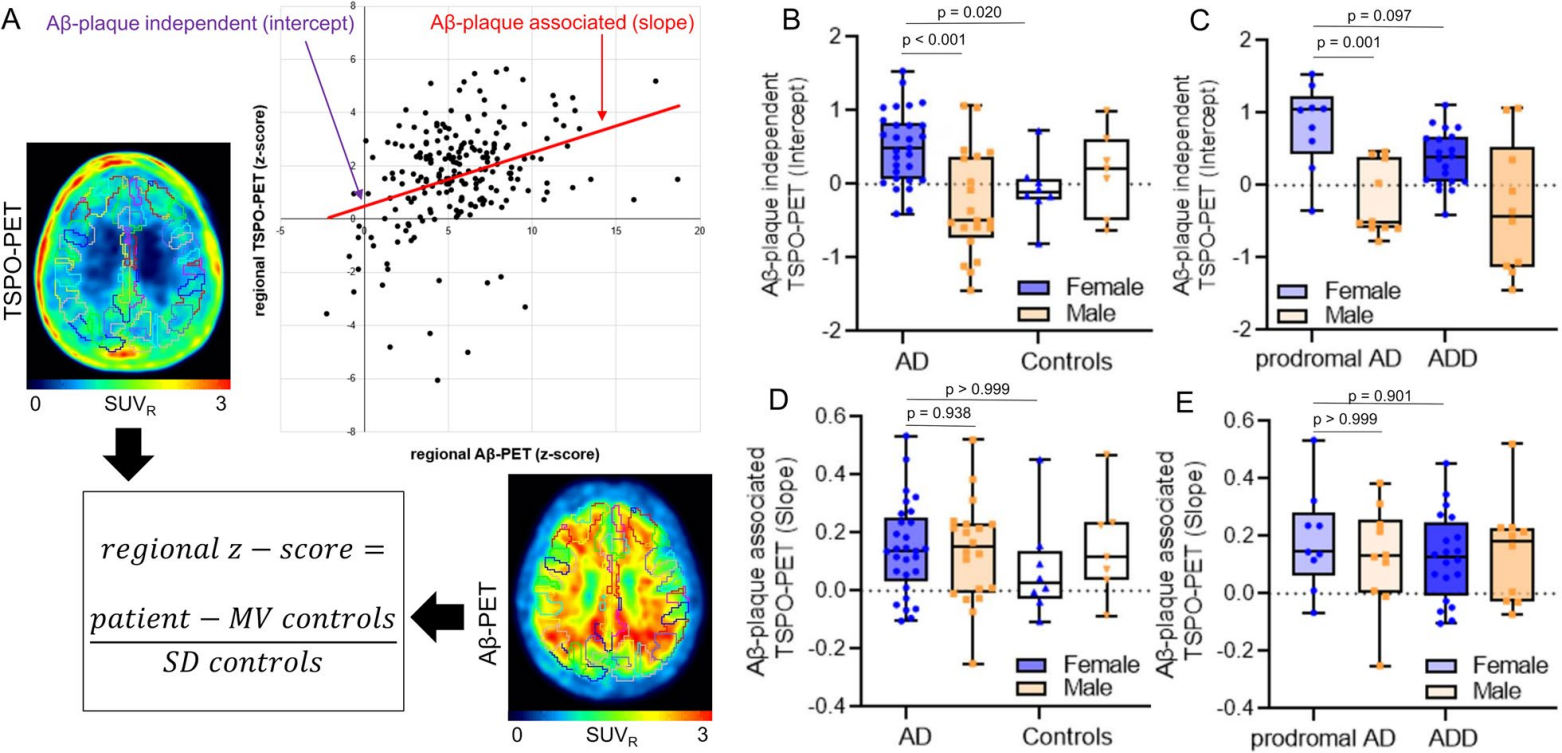
Impact of individual Aβ-plaque-independent and associated microglial response. **A** Cerebellum corrected standardized uptake value ratios (SUV_R_) were calculated for 210 cortical regions for TSPO-, Aβ- and Tau-PET. Per AD patient and brain region, the averaged regional increase (*z*-score) was calculated versus cognitively normal controls and plotted as a function of both tracers as shown. A two-dimensional microglia response index per patient was calculated, defined by the individual intercept and slope. **B** Comparison of Aβ-plaque-independent TSPO-PET (intercept) between female and male AD patients and cognitively normal controls and **C** prodromal AD and AD dementia (ADD). **D** Comparison of Aβ-plaque associated TSPO-PET (slope) between female and male AD patients and cognitively normal controls and **E** prodromal AD and ADD. P-values of the group comparisons derive from an ANCOVA with age, BMI and TSPO gene SNP as covariates, including FDR correction for multiple comparisons

Next, we investigated Aβ as the potential molecular trigger of higher TSPO-PET levels in females in more detail. To this end, we calculated a two-dimensional response index of microglial activation by assessment of TSPO- and Aβ-PET quantification in the parcellated brain. The TSPO-PET elevation in regions without elevated fibrillar Aβ (intercept) was defined as Aβ-plaque independent response and the association between regional TSPO- and Aβ-PET elevations was defined as the Aβ-plaque-dependent response (Fig. 2A).

Interestingly, only the Aβ-plaque-independent microglial response was higher in females with AD (intercept: 0.51 ± 0.11) when compared to males with AD (− 0.25 ± 0.13; *p* < 0.001) whereas the Aβ-plaque associated microglial response was indifferent between sexes (slope females with AD: 0.15 ± 0.03 vs. males with AD: 0.13 ± 0.04; *p* = 0.94; Fig. 2B, D), controlled for age, BMI and TSPO gene SNP. Since elevations of TSPO-PET binding were stage dependent in AD [17], we performed a dedicated analysis in prodromal and ADD patients. This phenotype-dependent analysis indicated the strongest Aβ-plaque-independent microglial response in females with prodromal AD (intercept: 0.74 ± 0.18) which was higher when compared to males with prodromal AD (intercept: − 0.23 ± 0.18, *p* = 0.001), but did not reach significance for the comparison of females and males with ADD (*p* = 0.097, Fig. 2C, E).

### Aβ-plaque-independent microglial response is associated with aggregated tau pathology in females

In the next step, we explored potential drivers of elevated Aβ-plaque-independent microglial response in females.

**Fig. 3:**
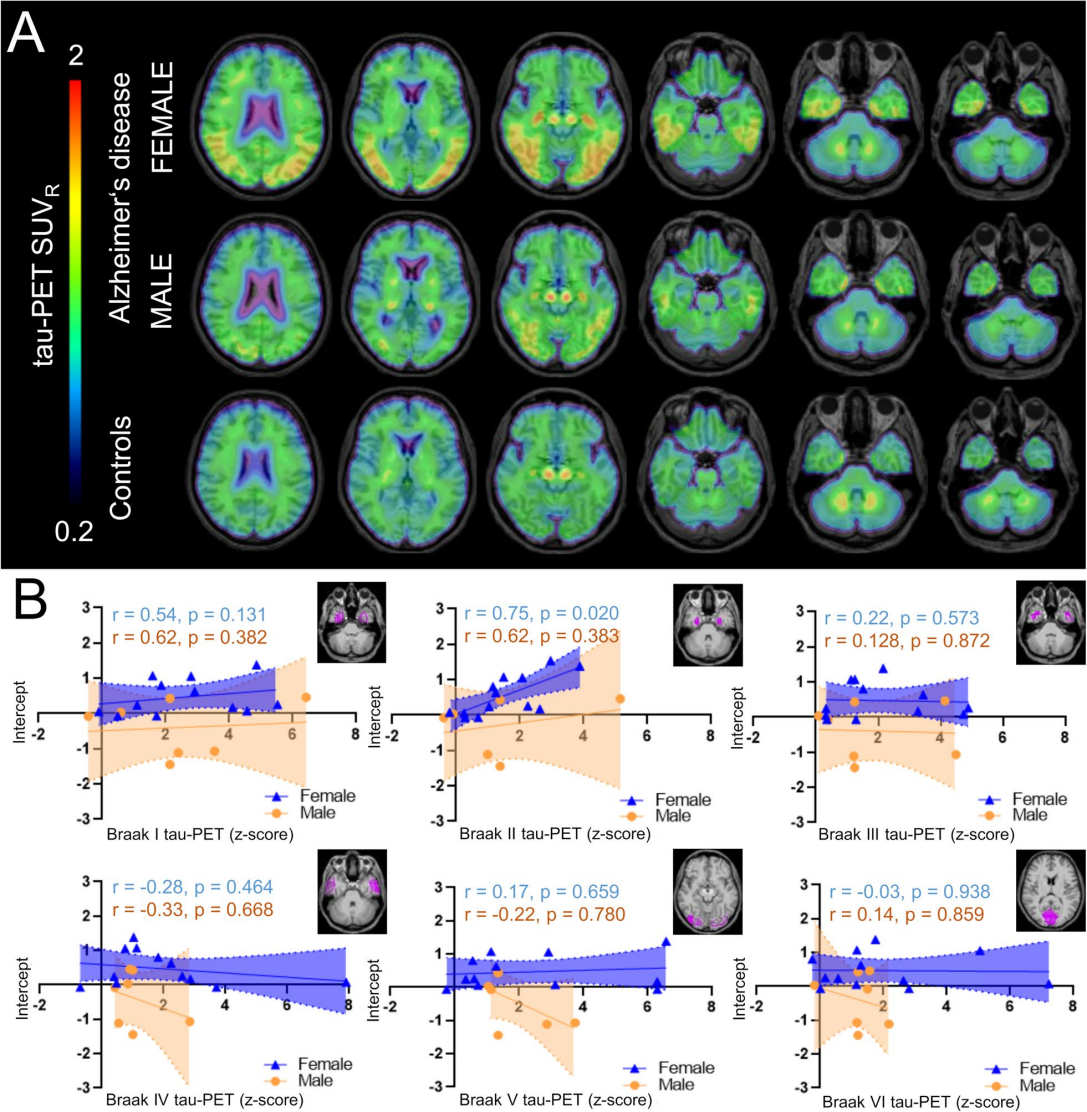
[^18^F]PI-2620 tau-PET binding in predefined target regions. **A** Group level binding intensity of [^18^F]PI-2620 tau-PET (cerebellum scaled standardized uptake value ratio, SUV_R_) for female and male AD patients and mixed sex cognitively normal individuals (CN), presented as axial overlays on a standard magnetic resonance imaging template. **B** Correlation of tau-PET z-scores for Braak-stage regions I–VI with the Aβ-plaque-independent microglial response (intercept) for females and males with AD. Anatomic regions are shown on an axial MRI atlas. AD female *n* = 14, AD male *n* = 10, CN mixed sex *n* = 5

Previously, we observed that regional tau pathology is stronger and more frequently associated with microglial activation when compared to regional Aβ-plaques [16]. Tau-PET was available in a subset of 24 AD patients (*n* = 14 female, *n* = 10 male) and 5 CN (*n* = 3 female, *n* = 2 male). Mean cortical tau-PET z-scores did not differ significantly between females and males with AD (females 1.89 ± 0.39 vs. males 0.90 ± 0.46; *p* = 0.12). The Aβ-plaque-independent microglial response was significantly associated with tau-PET z-scores in Braak-stage region II (*r* = 0.75, *p* = 0.020) in female AD patients, after controlling for age, BMI and TSPO gene SNP (Fig. 3). In male AD patients, no significant association between the Aβ-plaque-independent microglial response and tau-PET z-scores was observed (all *p* > 0.05, respectively) (Fig. 3). Static late-phase tau-PET quantification was validated against kinetic modeling of the full 60 min PET scan with image derived input function (IDIF) using the carotid artery (Additional file 1: Fig. S1). A strong agreement of tau PET SUVr *z*-scores with tau-PET VT was observed for all Braak-stage regions. The patterns of tau-PET VT reflected the patterns of tau-PET SUVr with strongest signal in AD-females, followed by AD-males and controls. Similar results were obtained for tau-PET patterns in female and male patients with AD compared to controls. We also tested if fluid biomarkers of Aβ (i.e., the soluble Aβ component) and tau pathology are associated with elevated Aβ-plaque-independent TSPO-PET signals in females and found no significant correlations for the Aβ_42/40_ ratio (*r* = 0.191, *p* = 0.43), *p*-tau-181 (*r* = − 0.095, *p* = 0.70) and the tau/Aβ index (*r* = − 0.318, *p* = 0.20). Additionally, the observed sex effects of Aβ-plaque dependent and independent microglia response were stable after controlling for the AD signature CSF markers (Aβ_42/40_ ratio, *p*-tau, tau/Aβ index) in a subset of the sample (intercept females with AD (*n* = 21): + 0.50 ± 0.14 vs. males with AD (*n* = 14): − 0.39 ± 0.18, *p* = 0.002; slope females with AD (*n* = 21): + 0.14 ± 0.04 vs. males with AD (*n* = 14): + 0.15 ± 0.05; *p* > 0.99).

### Sex moderates the relationship between obesity and plaque-independent microglial response

We then assessed clinical determinants of the different Aβ-plaque-independent microglial response in females and males.

**Fig. 4:**
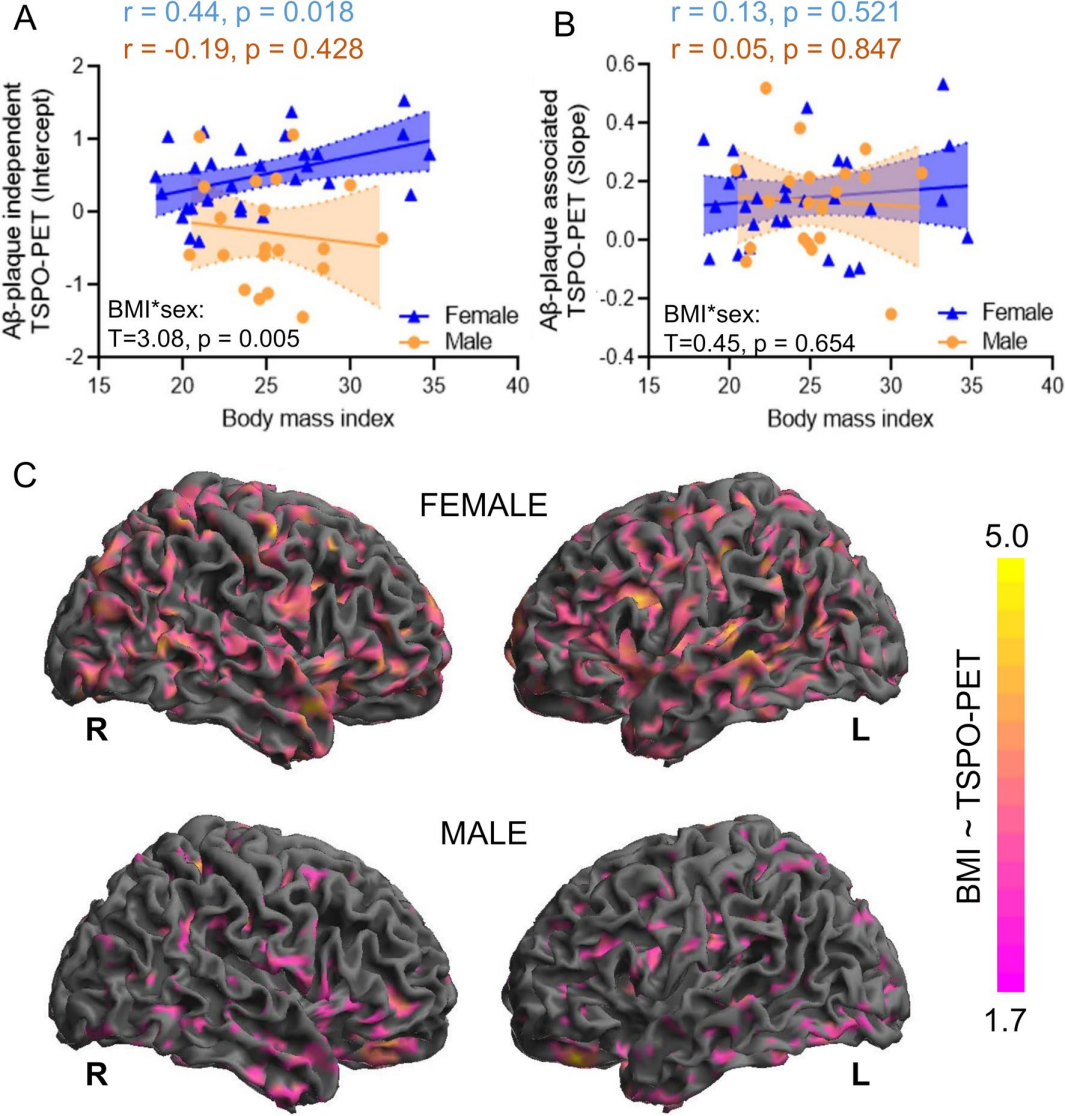
Associations of obesity with the indices of microglial response. **A** Correlation between the body mass index (BMI) and the Aβ-plaque-independent TSPO-PET signal (intercept) for females and males with AD. **B** Correlation between the BMI and the Aβ-plaque associated TSPO-PET signal (slope) for females and males with AD. **C** Voxel-wise association of BMI and TSPO-PET values as shown by surface projections for female and male AD patients after controlling for age and TSPO gene SNP using statistical parametric mapping (SPM, V12; significance threshold: topological FDR *p* < 0.05; cluster threshold: 50 voxels). AD female *n* = 29, AD male *n* = 20

Age and the Aβ-plaque-independent microglial response were not significantly associated in females (*r* = − 0.27, *p* = 0.17) and males with AD (*r* = − 0.03, *p* = 0.90) after adjustment for TSPO gene SNP and there was no significant age x sex interaction (*T* = 0.41, *p* = 0.69), when controlling for BMI, TSPO gene SNP and AD signature CSF markers. BMI and the Aβ-plaque-independent microglial response were significantly associated in females with AD (*r* = 0.44, *p* = 0.02) but not in males with AD (*r* = − 0.19, *p* = 0.44) after adjustment for TSPO gene SNP and there was a significant BMI x sex interaction (*T* = 3.08, *p* < 0.01; Fig. 4A), when controlled for TSPO gene SNP and AD signature CSF markers. No significant age x sex (*T* = 1.25, *p* = 0.22) or BMI x sex (*T* = 0.45, *p* = 0.65; Fig. 4B) interactions were observed with the Aβ-plaque associated microglial response. The stronger association of BMI and TSPO-PET in females was confirmed by a voxel-wise analysis. Here, the dedicated analysis in females with AD indicated 68,413 voxels with a positive association between BMI and TSPO-PET after controlling for age and SNP. Contrary, males with AD only showed 17,650 voxels with a positive association between BMI and TSPO-PET (Fig. 4C). The patterns of association were distributed across the whole brain without any emphasis on typical AD target regions.

## Discussion

In this study, we elucidate brain region-dependent sex differences of neuroinflammation in a detailed analysis of TSPO-PET signals in patients across the AD continuum. Our data show a sex-specific effect on the cortical TSPO-PET signal as an in vivo marker of microglia in female patients with AD, which notably was observed to be independent from the regional magnitude of fibrillar Aβ-plaque pathology.

To overcome regional heterogeneity of neuropathology distribution in AD [34], we applied a two-dimensional microglia response index to categorize the TSPO-PET signal into Aβ-plaque associated and Aβ-plaque-independent components of microglial activation by use of a multi-regional correlation analysis between TSPO-PET and Aβ-PET of the parcellated brain. This approach has two main advantages over classical target region-based analyses. First, the basal level of microglia activation can be obtained by the intercept of the regression, i.e., at a regional level of no fibrillar Aβ-plaque evidence. Second, the methodology is robust to atypical distribution of neuropathology in AD, since different peak regions in individual patients are reflected equally. This is important since asymmetry [35] or distinct regional accentuation [20, 34, 36] can also be present in a typical clinical phenotype. Thus, pooling patients with typical and atypical AD is feasible without inducing bias.

Similar to a recent multi-center TSPO-PET study in cognitively healthy men and women [11], we observed an effect of sex on the cortical TSPO-PET signal in our cohort, indicating that female patients with AD have higher TSPO-PET signals in frontal, temporal, parietal and posterior cingulate cortices as well as in Braak-stage II, IV and VI regions compared to male patients with AD. Our observations are supported by a growing number of clinical and preclinical studies [11, 37, 38], which also show sex-related differences in microglial quantification and distribution. One contributing factor to the observed effects could consist in female hormones, since estrogens play a major role in controlling microglial activity and TSPO [7]. While male rodents were shown to have overall more microglia early in postnatal development, females overtake later in development and in adulthood [38]. Not only microglial colonization of the brain, but also resting microglial configuration is sex dependent: male microglia have an enlarged soma, more reactiveness in physiological conditions, more pro-inflammatory responses and a higher migration capacity. Microglia of female mammalians on the other hand, have a higher phagocytic capacity and higher gene expression of cell repair and inflammatory control genes [39]. The observed sex-related differences in TSPO expression have potential impact when considering novel immunomodulatory therapies [40], since TSPO-PET signals predicted disease progression in 4-repeat tauopathies [41] and AD [42]. Thus, males and females with neurodegenerative diseases may deserve individualized treatment paradigms of immunomodulation at different disease stages.

Besides the observed sex differences in Aβ-plaque-independent microglia activation, we found that the regional reactivity of microglia to regional Aβ-plaque accumulation was not different between sexes. In addition, females comprised their strong Aβ-independent microglial response dominantly in prodromal AD. Assuming that Aβ mouse models mostly recapitulate prodromal AD, with evidence of Aβ accumulation but lack of tau aggregation, these findings are in line with recent rodent data from our lab [13]. Here, female *App*^*NL−G−F*^ mice showed a greater increase in TSPO-PET SUVr with age than did male *App*^*NL−G−F*^ mice, whereas there was no sex effect in fibrillar Aβ-accumulation as measured by [^18^F]florbetaben Aβ-PET in this mouse model. Another preclinical study concluded, that microglia in female mice tend to react earlier and in a more pronounced way than microglia in male mice [43]. It is further demonstrated, that female rodents better sustain injury after focal ischemia than male mice [44], possibly due to neurotrophic factors generated by microglia, that are beneficial for neuronal plasticity [45]. Besides, women with MCI have better performance in neuropsychological testing than males despite equivalent temporal lobe hypometabolism [46], which may imply an advantage of stronger microglia activation in response to early AD pathophysiology (i.e., soluble Aβ). In patients with AD, a significant effect of sex on tau-PET SUVrs in entorhinal and inferior temporal cortex as well as precuneus, corrected for regional Aβ-plaques was previously reported [47]. Beyond that, we find preliminary evidence of tau accumulation enhancing sex differences in Aβ-independent microglial activation in our female AD cohort. The transentorhinal region represents early stage tau accumulation as reported above [47], whereas tau in higher Braak-stage regions (IV–VI) was only detected in few patients in this early AD continuum cohort [17]. In order to fully evaluate influence of tau accumulation on the Aβ-independent microglial activation, a larger and more homogenous cohort of patients across Braak-stages is required. Neither tau-PET z-scores, nor tau CSF markers differed significantly between males and females with AD. Nonetheless, our data show a trend of higher tau deposition in females compared to males, which is also observed in some larger cohorts [48], which is one potential explanation of higher Aβ-plaque-independent microglial response in females compared to males. However, since previous studies could not determine a clear association of sex with regional tau in comparable clinically normal female and male individuals [49], tau may be a contributing factor but not the major driver of high Aβ-plaque-independent neuroinflammation in females with AD.

Moreover sex showed to moderate the relationship between obesity and microglial activation in patients with AD. Higher BMI was associated with higher Aβ-plaque-independent microglial response in females, but not in males. Furthermore, this association did not show a regional emphasis in typical AD target regions, speaking for a global brain effect without direct dependence from aggregation of misfolded Aβ or tau proteins. A recent PET-study in healthy volunteers likewise found a positive association between BMI and [^11^C]-PBR28 TSPO-PET SUVr [50]. A higher BMI in midlife predicted microglial activation 15 years later [50] as well as Aβ accumulation in the brain [51]. Multiple epidemiologic studies showed that chronic low-grade inflammation [52] and obesity [53] are associated with cognitive decline [50]. In addition, non-steroidal modulation of the central immune system has been proven to be anti-inflammatory effective in female mice of an AD model and showed associations with better cognitive performance [14], which confirms steroid- and consequently sex-hormone levels as an important disease mediator. In summary, our findings indicate that obesity is linked to high TSPO expression specifically in females with AD, which implies that high TSPO-PET levels deserve distinct interpretation among sexes. In view of personalized medicine, allocation for immunomodulatory treatments could require differentiation of sexes especially in obese individuals with AD.

Several limitations should be considered when interpreting the results of the manuscript. While the main attribution of the TSPO-PET signal is believed to derive from microglia, we note as a limitation that pro-inflammatory astrocytes also contribute to TSPO expression [54]. Furthermore, TSPO-PET may constitute an index of microglia abundance rather than an index of microglia activation in humans [55]. [^18^F]GE-180 has less penetration across the blood–brain barrier compared to other TSPO tracers [56] and since blood–brain barrier integrity can be disturbed in neurodegenerative disorders this may has an impact on tracer kinetics of our study [57]. However, PET to immunohistochemistry (CD68-positivity) correlations indicated that [^18^F]GE-180 TSPO-PET signals reflect the underlying microglia biology very well [58]. Furthermore, regional TSPO-PET signals were distinctly lower in participants with low-affinity binding status when compared to medium and high affinity binders, which made a priori exclusion necessary. Our study enrolled patients based on state-of-the-art clinical diagnosis but was not enriched by neuropathological confirmation. Thus, although the characterization of our patients was based on several biomarkers and detailed clinical work-up, misdiagnosed patients need to be considered as potential confounders in this study population. We used the cerebellum as a validated reference region for all three tracers ([^18^F]GE-180, [^18^F]flutemetamol and [^18^F]PI-2620) in order to ensure consistency in methodology. However, it needs to be considered that any tissue used for TSPO-PET ratios reflects a pseudo-reference region since microglia are abundant and activated throughout the brain. In this regard, the cerebellum emerged as a suitable pseudo-reference tissue for TSPO-PET imaging of AD [23].

As an outlook, there is growing evidence that tau pathology follows temporal accumulation of Aβ in AD patients [59] and that tau aggregation and microglial activation are closely associated [60]. Recent data showed that NLRP3 inflammasome activation triggers tau pathology [61] and, importantly, there is evidence that women present a higher level of tau pathology even at the preclinical stage of AD [49]. Thus, enhanced microglial activation in response to soluble Aβ could constitute the key factor and target to modulate elevated tau pathology in female AD patients.

## Conclusions

Sex has an impact on cortical TSPO-PET signals in patients with AD, indicating a stronger Aβ-plaque-independent component of TSPO-PET signal increases in females with prodromal AD when compared to males with prodromal AD. This sex difference in Aβ-independent microglial activation may be associated with tau accumulation. Furthermore, sex moderates the association between obesity and microglial activation, speaking for a dependence of obesity related TSPO enhancement from features that are present in females, as for example elevated tau. Strict adjustment for sex is required for TSPO-PET studies of microglial activation in human studies of neurodegenerative diseases. Further studies that address the mechanistic links between sex and TSPO expression are necessary to understand the ATN interplay, which forms the basis for personalized medicine.

### Supplementary Information

**Additional file 1: Table S1. **Detailed regional z-scores of TSPO-PET and Aβ-PET for female and male AD patients in six Braak-stage regions of interest and four amyloidosis regions of interest. CI =95% confidence interval. P –values show false discovery rate (FDR) corrected significance levels for the comparison of medium and high affinity binders (ANOVA). **Figure S1.** Validation of late-phase [^18^F]PI-2620 tau-PET quantification via carotid artery image derived input function (IDIF). Images show IDIF derived volume of distribution (VT) of [^18^F]PI-2620 tau-PET for female and male AD patients and mixed sex cognitively normal individuals, presented as axial overlays on a standard magnetic resonance imaging template. Plots show correlation of tau-PET z-scores for Braak-stage regions I–VI with tau-PET VT. AD female n = 13, AD male n = 9, cognitively normal mixed sex n = 3.

## Data Availability

The datasets used and/or analyzed during the current study are available from the corresponding author upon reasonable request.
