# Supplementary material for: Associations between sex, body mass index and the individual microglial response in Alzheimer’s disease
Source: J Neuroinflammation. 2024 Jan 23;21:30. doi: 10.1186/s12974-024-03020-y (PMC10804830; doi:10.1186/s12974-024-03020-y)
Supplement: Supplementary file 1 — Additional file 1: Table S1. Detailed regional z-scores of TSPO-PET and Aβ-PET for female and male AD patients in six Braak-stage regions of interest and four amyloidosis regions of interest. CI =95% confidence interval. P –values show false discovery rate (FDR) corrected significance levels for the comparison of medium and high affinity binders (ANOVA). Figure S1. Validation of late-phase [18F]PI-2620 tau-PET quantification via carotid artery image derived input function (IDIF). Images show IDIF derived volume of distribution (VT) of [18F]PI-2620 tau-PET for female and male AD patients and mixed sex cognitively normal individuals, presented as axial overlays on a standard magnetic resonance imaging template. Plots show correlation of tau-PET z-scores for Braak-stage regions I–VI with tau-PET VT. AD female n = 13, AD male n = 9, cognitively normal mixed sex n = 3. [file 12974_2024_3020_MOESM1_ESM.docx]

**Additional Methods**

**Image derived input function (IDIF)**

IDIFs were generated by automated extraction of the PET standard uptake value (SUV) signal from the carotid artery over the 60 minute dynamic PET scan.

For automated extraction of carotid artery SUV time series, dynamic PET images were first motion corrected using the implemented motion correction tool of PMOD (V4.2, PMOD Technologies, Zürich, Switzerland) by rigid alignment of subsequent frames. The resulting mean PET image was then warped to Montreal Neurology Institute (MNI) space via the 30-60 min summation image, using a custom in-house [^18^F]PI-2620 MNI template obtained by the PNEURO pipeline, via a high dimensional non-linear warping algorithm implemented in the Advanced Normalization Tools Software (ANTs) package. Independent component analysis (ICA) with a pre-defined 10 component solution was applied to the native space dynamic PET image in order to parcellate the image into variance components that represent maps of temporally correlated voxels. The underlying rationale is that voxels belonging to the carotid artery should show a highly temporally correlated SUV signal across the dynamic scan, which should be identifiable using ICA. The resulting component maps were warped to MNI space using the ANTs-derived high-dimensional warping parameters, and matched against a custom in-house carotid artery template in MNI space using spatial correlation to extract a subject-specific carotid component. The subject-specific carotid component in the MNI space was then automatically masked using a binary image that restricts the carotid artery to a segment in the upper part of the pars cervicalis, in line with the manual approach described above. Lastly, the masked subject-specific carotid image was warped back to native space using the ANTs derived warping parameters with nearest-neighbour interpolation to maintain a binary image. This image was further eroded using FSL to eliminate voxels close to the vessel walls, which may confound the carotid signal. The eroded binary carotid image was then applied to the native space dynamic PET image in order to extract the SUV time series (average value) across the 60 minutes scanning duration.

**Calculation of Volume of Distribution Images for tau PET**

Volume distribution (VT) images for [^18^F]PI-2620 tau PET were calculated with automatically segmented IDIFs using Logan Plots, which assume that the data become linear after an equilibration time t*. t* was fitted based on the maximum error criterion, which indicates the maximum relative error between the linear regression and the Logan-transformed measurements in the segment starting from t*. The maximum error was set to 10 %. The percent masked pixels were set to 0 %. The Putamen, which was defined by manual placement of a VOI (sphere with a diameter of 10 mm), served as tissue region. Tau PET VTs were correlated against z-scores of late static tau PET standardized uptake value ratios (SUVr) as a validation experiment.

**Additional Results**

**Table S1**

| **PET** | **AD female** | | | **AD male** | | |  |
| --- | --- | --- | --- | --- | --- | --- | --- |
| **TSPO-PET z-scores** | **MAB (mean, 95%-CI)** | **HAB (mean, 95%-CI)** | **P** MAB/HAB **(FDR)** | **MAB (mean, 95%-CI)** | **HAB (mean, 95%-CI)** | **P** MAB/HAB **(FDR)** |  |
| Braak I | 1.105 (0.544 - 1.666) | 1.488 (0.818 - 2.158) | 0.948 | 0.492 (-0.567 - 1.552) | 0.162 (-0.792 - 1.116) | 0.798 |  |
| Braak II | 1.126 (0.610 - 1.642) | 0.660 (0.044 - 1.276) | 0.823 | 0.576 (-0.307 - 1.458) | -0.123 (-0.918 - 0.672) | >0.999 |  |
| Braak III | 1.646 (1.253 – 2.039) | 1.627 (1.158 - 2.096) | 0.951 | 1.133 (0.178 – 2.088) | 1.468 (0.607 - 2.328) | 0.853 |  |
| Braak IV | 0.879 (0.596 - 1.198) | 0.918 (0.559 - 1.278) | >0.999 | 0.257 (-0.381 - 0.894) | 0.682 (0.108 - 1.256) | >0.999 |  |
| Braak V | 1.340 (0.676 - 2.004) | 0.705 (-0.088 - 1.497) | >0.999 | 0.539 (-0.187 - 1.266) | 0.909 (0.255 - 1.563) | 0.890 |  |
| Braak VI | 0.908 (0.417 – 1.400) | 0.137 (-0.449 - 0.724) | 0.500 | -0.112 (-0.798 - 0.574) | -0.157 (-0.774 – 0.461) | 0.921 |  |
| Frontal cortex | 1.148 (0.818 - 1.478) | 1.257 (0.863 – 1.651) | 0.835 | 0.257 (-0.480 - 0.995) | 0.828 (0.164 - 1.493) | >0.999 |  |
| Temporal cortex | 0.749 (0.438 - 1.061) | 0.903 (0.531 - 1.275) | 0.749 | 0.098 (-0.645 - 0.840) | 0.298 (-0.370 - 0.966) | 0.759 |  |
| Parietal cortex | 1.013 (0.672 - 1.355) | 0.821 (0.414 - 1.229) | 0.780 | 0.242 (-0.427 - 0.911) | 0.544 (-0.059 - 1.146) | 0.828 |  |
| PCC/Precuneus | 1.228 (0.761 - 1.694) | 0.957 (0.400 - 1.514) | 0.908 | 0.143 (-0.622 - 0.907) | 0.631 (-0.058 - 1.319) | 0.850 |  |
| **Aβ-PET z-scores** | **MAB (mean, 95%-CI)** | **HAB (mean, 95%-CI)** | **P** MAB/HAB **(FDR)** | **MAB (mean, 95%-CI)** | **HAB (mean, 95%-CI)** | **P** MAB/HAB **(FDR)** |  |
| Braak I | 2.446 (1.768 - 3.125) | 1.694 (0.883 - 2.504) | 0.527 | 1.563 (0.007 - 3.119) | 2.251 (0.850 - 3.652) | 0.631 |  |
| Braak II | 0.832 (0.301 - 1.363) | 0.726 (0.092 - 1.359) | 0.883 | 0.576 (-0.529 - 1.681) | 0.508 (-0.488 - 1.503) | 0.925 |  |
| Braak III | 3.131 (2.405 - 3.856) | 2.546 (1.680 - 3.412) | 0.600 | 2.584 (1.085 - 4.083) | 3.500 (2.150 - 4.850) | >0.999 |  |
| Braak IV | 5.906 (4.953 - 6.859) | 5.291 (4.153 - 6.428) | 0.579 | 5.238 (3.344 - 7.132) | 6.356 (4.650 - 8.061) | 0.943 |  |
| Braak V | 2.702 (1.837 - 3.566) | 2.668 (1.636 - 3.700) | 0.960 | 2.985 (1.769 - 4.200) | 3.501 (2.406 - 4.595) | 0.580 |  |
| Braak VI | 4.561 (3.294 - 5.829) | 3.265 (1.752 - 4.777) | 0.478 | 3.829 (1.832 - 5.827) | 4.930 (3.131 - 6.729) | 0.680 |  |
| Frontal cortex | 6.683 (5.706 - 7.660) | 5.552 (4.386 - 6.718) | 0.705 | 5.008 (3.406 - 6.610) | 6.489 (5.047 - 7.932) | >0.999 |  |
| Temporal cortex | 5.562 (4.602 - 6.521) | 4.822 (3.677 - 5.968) | 0.537 | 4.829 (3.072 - 6.585) | 5.630 (4.048 - 7.212) | 0.703 |  |
| Parietal cortex | 6.021 (4.988 - 7.054) | 5.407 (4.174 - 6.640) | 0.554 | 5.089 (3.564 - 6.613) | 5.970 (4.597 - 7.343) | 0.772 |  |
| PCC/Precuneus | 9.266 (8.074 - 10.457) | 7.246 (5.823 - 8.668) | 0.350 | 7.833 (5.681 – 9.986) | 9.115 (7.177 - 11.053) | >0.999 |  |

**Table S1:** Detailed regional z-scores of TSPO-PET and Aβ-PET for female and male AD patients in six Braak-stage regions of interest and four amyloidosis regions of interest. CI =95% confidence interval. P –values show false discovery rate (FDR) corrected significance levels for the comparison of medium and high affinity binders (ANOVA).

**Figure S1**


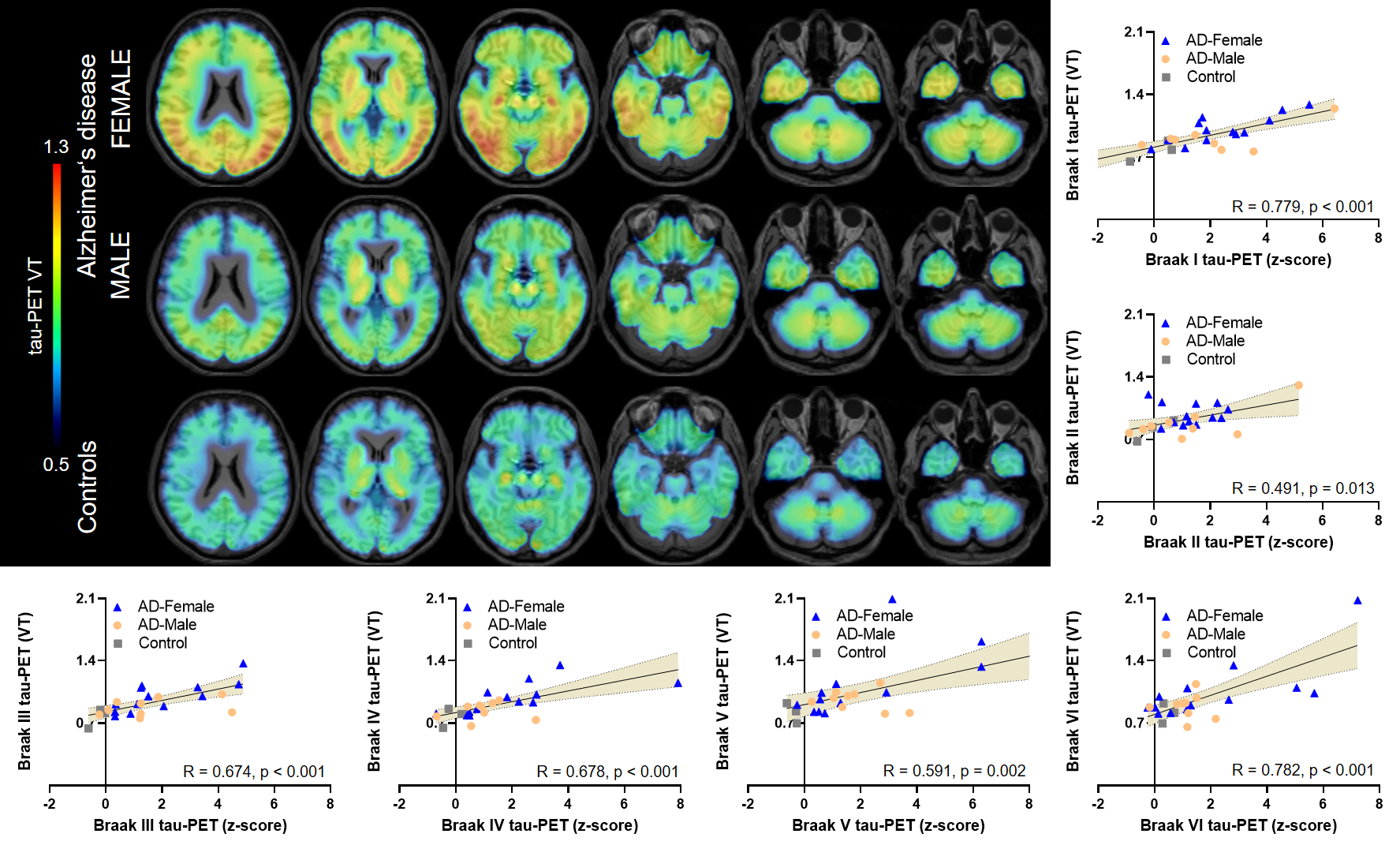


**Figure S1: Validation of late-phase** **[^18^F]PI-2620 tau-PET quantification via carotid artery image derived input function (IDIF).** Images show IDIF derived volume of distribution (VT) of [^18^F]PI-2620 tau-PET for female and male AD patients and mixed sex cognitively normal individuals, presented as axial overlays on a standard magnetic resonance imaging template. Plots show correlation of tau-PET z-scores for Braak-stage regions I-VI with tau-PET VT. AD female n = 13, AD male n = 9, cognitively normal mixed sex n = 3.
